# Interventional Effect of Donkey Bone Collagen Peptide Iron Chelate on Cyclophosphamide Induced Immunosuppressive Mice

**DOI:** 10.3390/nu16152413

**Published:** 2024-07-25

**Authors:** Xiang-Rong Cheng, Zi-Wei Zhao, Yu-Yao Chen, Jie Song, Jia-Hui Ma, Chen-Xi Zhang, Issoufou Amadou, Nai-Yan Lu, Xue Tang, Bin Guan

**Affiliations:** 1 School of Food Science and Technology, Jiangnan University, Wuxi 214122, China; 2 State Key Laboratory of Food Science and Resources, Jiangnan University, Wuxi 214122, China; 3 Faculty of Agriculture and Environment Sciences, Dan Dicko Dankoulodo University of Maradi, Maradi BP 465, Niger; 4 Department of Pharmacy, Affiliated Wuxi Fifth Hospital of Jiangnan University, Wuxi 214011, China; 5 Department of Pharmacy, The Fifth People’s Hospital of Wuxi, Wuxi 214011, China

**Keywords:** donkey bone, collagen peptide, iron chelate, immunosuppression, cyclophosphamide, purine salvage pathway

## Abstract

Immunodeficiency can disrupt normal physiological activity and function. In this study, donkey bone collagen peptide (DP) and its iron chelate (DPI) were evaluated their potential as immunomodulators in cyclophosphamide (Cytoxan^®^, CTX)-induced Balb/c mice. The femoral tissue, lymphocytes, and serum from groups of mice were subjected to hematoxylin and eosin (H&E) staining, methylthiazolyldiphenyl-tetrazolium bromide (MTT) cell proliferation assays, and enzyme-linked immunosorbent assay (ELISA), respectively. Furthermore, a non-targeted metabolomics analysis based on UPLC–MS/MS and a reverse transcription polymerase chain reaction (RT-qPCR) technology were used to explore the specific metabolic pathways of DPI regulating immunocompromise. The results showed that CTX was able to significantly reduce the proliferative activity of mouse splenic lymphocytes and led to abnormal cytokine expression. After DP and DPI interventions, bone marrow tissue damage was significantly improved. In particular, DPI showed the ability to regulate the levels of immune factors more effectively than Fe^2+^ and DP. Furthermore, metabolomic analysis in both positive and negative ion modes showed that DPI and DP jointly regulated the levels of 20 plasma differential metabolites, while DPI and Fe^2+^ jointly regulated 14, and all 3 jointly regulated 10. Fe^2+^ and DP regulated energy metabolism and pyrimidine metabolism pathways, respectively. In contrast, DPI mainly modulated the purine salvage pathway and the JAK/STAT signaling pathway, which are the key to immune function. Therefore, DPI shows more effective immune regulation than Fe^2+^ and DP alone, and has good application potential in improving immunosuppression.

## 1. Introduction

The human immune system plays a crucial role in defending against external pathogens and combating various disease states, and it is involved in regulating the various stages of injury healing in the body [1]. Whenever it is disturbed by environmental factors, drugs, chronic diseases, etc., immune organs and cells in the immune system can be damaged to varying degrees, leading to immune deficiency [2]. Nowadays, medical therapy and immune replacement therapy are common strategies for treating immune deficiency, but these interventions may lead to dysfunction of cytokines in the body, disruption of the gut microbiota balance, induction of inflammatory reactions, and the triggering of allergies [3,4]. However, recent research indicates that natural immune modulators with functional activity have the potential to enhance immune responses, improve malnutrition, and regulate metabolic imbalances Therefore, compared with medical therapy, the intervention of immunocompromised patients from dietary nutrients has the characteristics of a mild effect and high-level safety, and offers a broad development prospect [5,6,7].

Building on the understanding of the immune system’s susceptibility to various disturbances and the limitations of conventional treatments, the exploration of natural immune modulators has opened new avenues for immune support. Immunomodulatory peptides derived from animal sources stand out as essential elements of dietary immune modulators, demonstrating the ability to harmoniously regulate immune responses across both innate and adaptive immunity. These peptides are particularly noteworthy for their benign nature, characterized by the absence of toxic side effects, their cost-effectiveness, and their substantial value for further development. As animal husbandry advances, a large number of by-products, such as carcass residues, are generated during animal processing, which brings abundant poultry and livestock bone resources. These bones are rich in collagen, accounting for 80–90% of their total protein composition [8]. Notably, research has indicated that collagen peptides derived from these sources exert specific immune regulatory effects. This efficacy may be attributed to the high proline content of these peptides, which is instrumental in preserving their structural and functional integrity throughout the digestive process [9]. Furthermore, collagen peptides with a small molecular weight have been reported to possess strong immune activity [10].

Donkey bones are commonly discarded as by-products of the donkey industry, resulting in the waste of donkey bone resources and contributing to environmental pollution. The rich collagen and peptide products found in donkey bones exhibit high biological activity and that have been demonstrated to possess antioxidant and protective functions against cellular damage [8]. Furthermore, previous studies have indicated that the traditional Chinese food as medicine, Ejiao (*Colla Corii Asini*), and its peptide–iron (EPI) chelate can effectively enhance the hematopoietic function of mice, protect damaged organs, and exhibit intestinal immune function [11]. The main component of Ejiao is donkey skin, which is highly similar in amino acid composition to donkey bone. Thus, it’s hypothesized that donkey collagen peptide (DP) and its iron chelation (DPI) have the function of regulating immune potential.

This study aimed to further study the donkey collagen peptide, its iron chelation immune activity, and its regulation mechanisms. In the present study, peptides derived from donkey bone and their iron chelates were administered to cyclophosphamide (Cytoxan^®^, CTX)-induced mice to evaluate the immunomodulatory potential of these compounds. On the basis of the analysis of immune organs, cells, and factors, LC-MS non-targeted metabolomics technology was also used to explore the impact on the metabolic pathways of the immunodeficiency state, so as to reveal the possible immune regulatory mechanism.

## 2. Materials and Methods

### 2.1. Materials and Reagents

Donkey bone meal was purchased from Dong-E-E-Jiao Co., Ltd., Liaocheng, China. CTX (PHR1404) was purchased from Sigma Aldrich, Shanghai, China. Levamisole was purchased from Shandong Renhetang Pharmaceutical Co., Ltd., Linyi, China. Papain (≥800 U/mg) and dialysis bags (3500 Da) were purchased from Shanghai Yuanye Biotechnology Co., Ltd., Shanghai, China. Tumor necrosis factor alpha (TNF-α), interleukin-3 (IL-3), interleukin-6 (IL-6), and interferon gamma (IFN-γ) assay kits were purchased from Xiamen Huijia Biotechnology Co., Ltd., Xiamen, China. The target gene primers were purchased from Jinweizhi (Suzhou) Biotechnology Co., Ltd., Suzhou, China.

### 2.2. Preparation of the DPI

Following a 24-h degreasing process using petroleum ether, the donkey bone collagen underwent a series of purification steps. It was first decalcified with a 0.8 M HCl solution for 12 h, then subjected to salting out with a Na_2_SO_4_ solution for another 12 h. Subsequently, the collagen was dialyzed against a 0.1 M acetic acid solution for a full 24 h to ensure thorough removal of impurities. Finally, to complete the purification, the collagen was dialyzed with ultrapure water for an extended period of 48 h. After these meticulous steps, the donkey bone collagen was successfully freeze-dried, yielding a high-quality freeze-dried product. Donkey bone collagen is initially dissolved in distilled water, after which the pH is adjusted to neutral at 7.0 and the temperature is gently raised to 50 °C. At this stage, papain is introduced for enzymatic hydrolysis at a precise concentration of 750 U/mL for a period of 3 h. Once the hydrolysis process is complete, the solution is subjected to boiling for 15 min to effectively inactivate the enzyme. Subsequently, the mixture is allowed to cool down to room temperature and then centrifuged at 4 °C at a speed of 6000 rpm for 20 min to separate the components. The supernatant is filtered through a 0.45 μm microporous membrane, resulting in the acquisition of DP. The subsequent preparation of DPI is executed according to the well-established techniques of our research group, as previously reported [12], and, thus, the specific details are not reiterated here for the sake of conciseness.

### 2.3. Animals and Treatments

Fifty 6-week-old male Balb/c mice were purchased from Beijing Weitong Lihua Experimental Animal Technology Co., Ltd., Beijing, China, with the license number SYXK (SU) 2021-0056. The mice were raised in an SPF-level barrier environment, with a controlled temperature of 23–27 °C and a relative humidity of 40–60%, under an artificial light/dark cycle for 12 h. Animal experiments were approved by the Experimental Animal Welfare and Ethics Review Committee of Jiangnan University, with the serial number *JN.No20230515b0500620[166]*.

Following a one-week adaptation period, the mice were randomly divided into six groups: the normal control group (CON, *n* = 8), cyclophosphamide group (CTX, *n* = 8), levamisole-positive control group (LMS, *n* = 8), FeSO_4_ group (FE, *n* = 8), donkey collagen peptide group (DP, *n* = 9), and donkey collagen peptide iron chelate group (DPI, *n* = 9). Except for the CON group receiving an equal volume of saline intraperitoneal injection every day, all other groups received 100 μL injections per day of CTX (80 mg/kg, diluted with saline) for a total of 3 days [13]. After 3 days, CON and CTX groups were given 200 μL normal saline by gavage, while 200 μL of the corresponding intervention agents were given to the LMS, FE, DP, and DPI groups, for 12 days, and body weight was recorded every 3 days. The peptide and Fe^2+^ dosages were determined based on Wu [14] and Cheng [15], with moderate doses of peptide (1.2 g/kg) and iron (2.0 mg/kg) solutions selected for intervention. Chelation was carried out according to the above peptide and iron doses to obtain the peptide iron chelate required for the DPI group. On the 16th day, the mice were fasted overnight and anesthetized with isoflurane inhalation after weighing. They were euthanized using the cervical dislocation method, and then immediately dissected. Organ samples were collected on ice and weighed, and plasma, serum, and organ tissues were frozen at −80 °C.

### 2.4. Determination of Whole Blood Hemogram Indexes

50 μL of blood was collected from the orbital sinuses of mice and collected in heparinized tubes for the detection of basic blood indexes. The number of white blood cells (WBC) and red blood cells (RBC) and the proportion of neutrophils (Neu), eosinophils (Eos), basophils (Bas), monocytes (Mon), lymphocytes (Lym), and platelets (PLT) were measured using an animal automatic hematology analyzer.

### 2.5. Histopathological Examination

Following euthanasia, the femur was dissected from the mouse and the fascia and fragmented meat were carefully removed. The entire femur was then immersed in 4% paraformaldehyde for fixation, which was maintained for 24 h. Subsequently, the femur was transferred to a 10% EDTA solution for decalcification, with the solution being replaced every two days until the bone became soft enough to be punctured, a process that took a total of 14 days [16]. After fixation, the tissue underwent paraffin embedding, and thin sections were prepared using a pathological slicer. Hematoxylin and eosin (H&E) staining was performed, and the stained sections were observed under an optical microscope [17].

### 2.6. Determination of Splenic Lymphocyte Proliferation Ability

In brief, a portion of the mouse spleen was collected in a sterile environment to prepare a splenic lymphocyte suspension. The proliferation ability of mouse spleen lymphocytes induced by concanavalin A (ConA) was detected using methylthiazolyldiphenyl-tetrazolium bromide (MTT) assay [18]. Lymphocytes were inoculated in 96-well plates at a density of 200 cells per well (210 μL total volume). The blank control group received 210 μL of RPMI-1640 complete medium, while the ConA group received 10 μL of ConA solution (100 μg/mL) and 10 μL of RPMI-1640 complete culture medium without an inducer. After incubation, 20 μL of MTT solution was added to each well, and the plates were incubated for an additional 4 h. The plates were then centrifuged at 1800 rpm for 5 min, and the supernatant was carefully discarded. Next, 150 μL of DMSO was added to each well, and the plates were shaken for 10 min. Finally, the absorbance values of each well at a wavelength of 570 nm were measured using a microplate enzyme-linked immunosorbent assay (ELISA) reader to determine the relative number of cells. The optical density (OD) value of each pore was measured with an enzyme-labeled instrument at 570 nm wavelength, and the stimulation index (SI) was used as an evaluation index to reflect the proliferation ability of lymphocytes. The stimulation index was calculated according to Formula (1), as follows:

(1) $$\mathrm{SI}(\%)=\frac{OD\;sample-OD\;vehicle}{OD\;control-OD\;vehicle}\times 100\%$$

### 2.7. Determination of Cytokines in Serum

The levels of IL-3, IL-6, TNF–α, and IFN-γ in serum were measured. The determination of cytokines was conducted using an ELISA assay kit (Xiamen Huijia Biotechnology Co., Ltd., Xiamen, China). Enzyme-labeled reagents were added to a 96-well plate according to the instructions, followed by incubation and washing steps. After the color reaction, 1.0 M H_2_SO_4_ solution was added to stop the reaction. Immediately thereafter, the absorbance values of each well were measured at a wavelength of 450 nm, and the cytokine concentration was calculated based on the obtained standard curve equation.

### 2.8. Real-Time Quantitative PCR

RNA was extracted from thymus tissue using a column extraction method according to the instructions. Total RNA was then reverse-transcribed into cDNA using the HiScript^®^ III RT SuperMix reverse transcription kit (Vazyme Biotechnology Co., Ltd., Nanjing, China). Quantitative real-time reverse transcription polymerase chain reaction (RT-qPCR) amplification was performed using the 2× Taq Pro Universal SYBR qPCR Master Mix. The β-actin was used as an endogenous control for normalizing target gene mRNA expression. The relative expression levels of target genes were calculated using the 2^−ΔΔCT^ method. The primer sequences are listed in Table 1.

### 2.9. Untargeted Metabolomics Analysis for Plasma Based on UPLC–MS/MS

Metabolomics analysis and data processing were carried out according to previous reports [19]. In this study, we adopted standardized sample processing to ensure the accuracy of the analysis of the plasma samples. We referred to Cheng [15] for a method of plasma samples pretreatment and to set the LC–MS/MS parameters.

### 2.10. Statistical Analysis

SPSS 23 software was employed to perform a one-way analysis of variance (ANOVA) and Duncan’s multiple comparison analysis on the data. The experimental data were expressed as mean ± standard deviation (SD). Significant differences were considered at *p* < 0.05, indicating statistical significance.

MS raw data were obtained using the software Xcalibur (version 3.1, Thermo Scientific, Waltham, MA, USA). The data underwent denoising, baseline correction, and normalization using the total peak area method to generate the modeling standard matrix. Multivariate statistical analysis was performed on the data using the SIMCA-P 13.0 software package, employing principal component analysis (PCA) and orthogonal partial least squares discriminant analysis (OPLS-DA) to screen for intergroup differential metabolites. Differential metabolites were then matched and identified against the Human Metabolome Database (HMDB) using the supplier software Compound Discoverer 3.3. Differential metabolic pathway analysis was conducted using the MetaboAnalyst 6.0 website [20].

## 3. Results

### 3.1. The Impact of DPI on Body Weight and Organ Index

**Figure 1:**
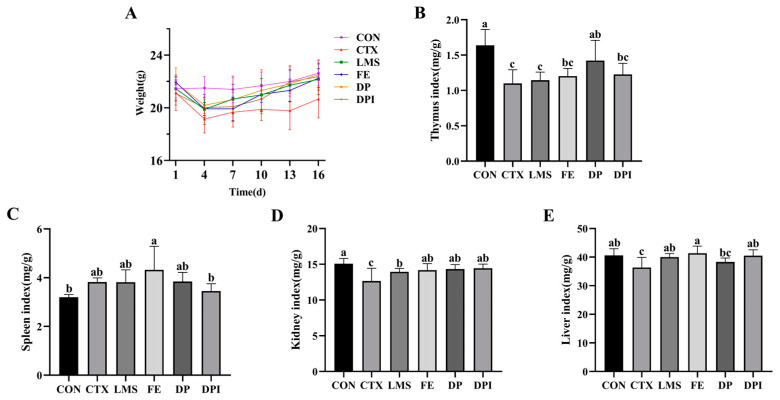
Changes in body mass of mice and organ index of mice. Normal control group (CON), cyclophosphamide group (CTX), levamisole-positive control group (LMS), FeSO_4_ group (FE), donkey collagen peptide group (DP), and donkey collagen peptide iron chelate group (DPI). Effect of DPI on body weight (**A**), thymus index (**B**), spleen index (**C**), kidney index (**D**), and liver index (**E**) in immunocompromised mice. Different lowercase letters indicate a significant difference between different groups (*p* < 0.05).

Body weight changes in mice in each group were monitored during the feeding period (Figure 1A). A significant reduction in body weight was observed following a 3-day continuous intraperitoneal injection of CTX in Balb/c mice (*p* < 0.01). The body weight of mice in each intervention group increased significantly (*p* < 0.05). Notably, the DPI group exhibited the most significant regulatory effect, with body weights approaching the normal levels seen in the CON group. In contrast, the FE group exhibited a slower regulatory response with a noticeable lag. These findings suggest that DPI has a moderating effect on weight loss induced by CTX in immunocompromised mice.

After the intervention, the organ indices of each group of mice were evaluated (Figure 1B–E). Following CTX administration, the spleen index showed a non-significant increase (*p* ≥ 0.05) (Figure 1C), while the liver index, thymus index, and kidney index significantly decreased (*p* < 0.05) (Figure 1B,D,E). The intervention effect from each treatment group did not significantly affect the spleen index, with only the DPI group showing an altered spleen index. The DP intervention significantly decreased the thymic index of mice (*p* < 0.05) to normal levels. The intervention effects on the liver index and kidney index in each treatment group were relatively similar, with a significant increase compared to the CTX group (*p* < 0.05). These findings demonstrated the protective effect of DPI on immune organs.

### 3.2. The Effect of DPI on Peripheral Blood Count

**Figure 2:**
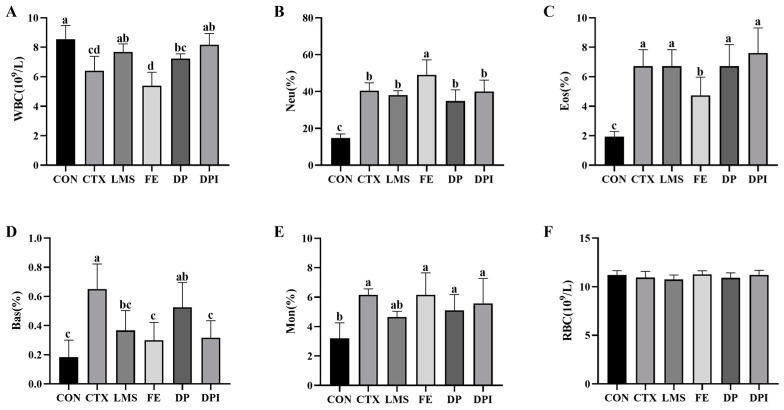
Changes in the peripheral blood count of mice. (**A**) The number of WBCs, (**B**) neutrophil ratio, (**C**) eosinophil ratio, (**D**) basophils ratio, (**E**) monocyte ratio, and (**F**) the number of RBCs. White blood cells (WBCs), red blood cells (RBCs), the proportion of neutrophils (Neu), eosinophils (Eos), basophils (Bas), and monocytes (Mon). Different lowercase letters indicate a significant difference between different groups (*p* < 0.05).

On the third day of CTX administration, blood count indicators for each group of mice were measured to evaluate the immunosuppressive effect of CTX, based on the levels of WBC, RBC, and PLT (Table 2). It was observed that a 3-day continuous intraperitoneal injection of CTX significantly reduced the levels of WBCs and RBCs in the peripheral blood of mice (*p* < 0.05), with no significant effect on PLT levels, consistent with the results observed by Sun et al. [21]. Figure 2 showed that after 14 days of intervention, there were no significant differences in erythroid parameters among the mouse groups, which may be due to the natural recovery and compensatory mechanisms of the body. Compared with the CTX group, the LMS group somewhat reduced the number of WBCs and the proportion of Bas (*p* < 0.05). The FE group adjusted the Eos ratio and Bas ratio (*p* < 0.05), but it further imbalanced the WBC number, Neu ratio, and Mon ratio levels. The effects of DP and DPI were similar, both reducing the number of WBCs and the proportion of Bas to some extent, but the effect of DPI was more significant (*p* < 0.05). These results indicated that DPI comprehensively mitigated the destructive effect of CTX on peripheral blood, particularly evident in the white blood cell parameters.

### 3.3. The Effect of DPI on the Pathology of Bone Marrow Tissue

**Figure 3:**
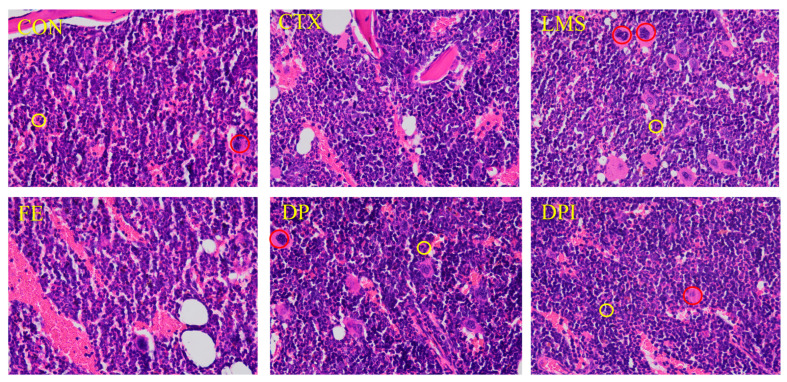
Histological structure of femur bone marrow of mice in each group (H&E staining, 400×).

The H&E staining histopathological image of the femoral bone marrow (Figure 3) revealed that the internal cells in the CON group were tightly and neatly arranged, with a clear tissue structure. Well-developed and mature megakaryocytes (red circle) and Neu (yellow circle) were evident. The CTX group showed vacuolar degradation, deformation of megakaryocytes, uneven distribution, and severe fibrosis, indicating that CTX significantly impacted bone marrow structure and immune function, resulting in a degree of bone marrow suppression. Each administration group exhibited a certain degree of intervention effect, but the extent of recovery varied. In the LMS group, megakaryocyte morphology returned to normal, fibrosis decreased, and some cavities remained. The FE group showed significant vacuolar degradation, low recovery of nucleated cells, severe fibrosis, and a poor improvement effect on the femoral structure and bone marrow cell levels. The DP group significantly improved the megakaryocyte morphology, with tightly arranged cells and fewer cavities. The DPI group significantly enhanced the pathological damage to the bone marrow, with evenly distributed and tightly arranged cells, no cavities, and clear and visible structures. CTX disrupted the functional structure of bone marrow, leading to the destruction of lymphocyte populations and immune system damage. LMS, DP, and DPI effectively alleviated the structural damage, with DPI being the most effective.

### 3.4. The Effect of DPI on the Proliferation Activity of Splenic Lymphocytes

**Figure 4:**
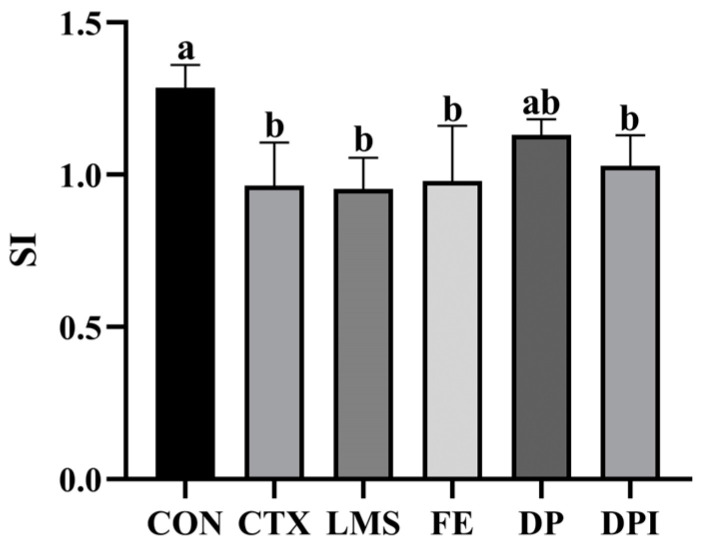
Proliferation of splenic lymphocytes in mice. Stimulation index (SI). Different lowercase letters indicate a significant difference between different groups (*p* < 0.05).

As shown in Figure 4, compared with the CON group, the proliferation of splenic lymphocytes in the CTX group was significantly reduced (*p* < 0.05), indicating the inhibition and killing of splenic T cells by CTX. The splenic lymphocyte stimulation index (SI) of the DP group showed a similar level to that in CON group, with the DPI group following closely, suggesting that DP most effectively improved splenic lymphocyte proliferation in CTX-induced immunosuppressive mice (*p* ≥ 0.05). The intervention effects of other treatment groups on splenic lymphocyte proliferation ability were not significant (*p* ≥ 0.05). It suggested that the administration of DPI and DP may result in immune enhancement.

### 3.5. The Regulatory Effect of DPI on the Apoptosis Pathway of Thymic Tissue Cells

**Figure 5:**
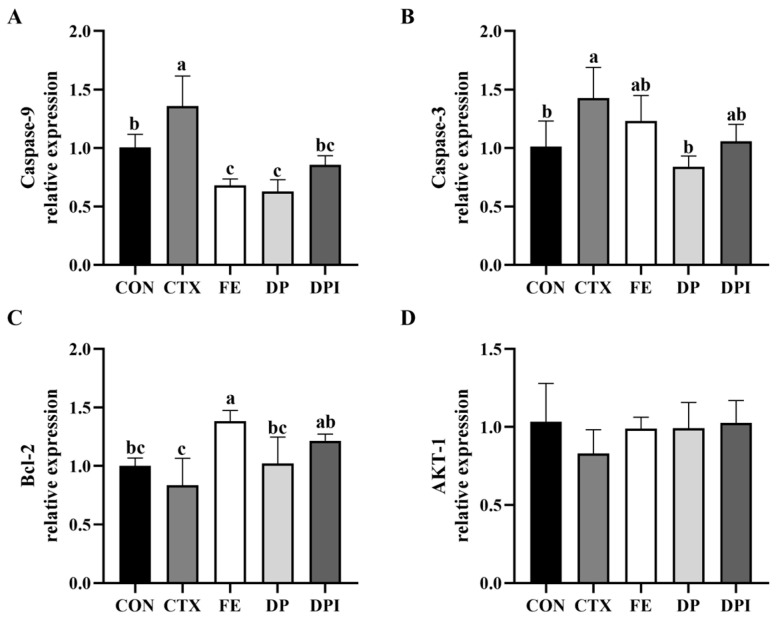
Effect of donkey bone collagen peptide iron chelate on the expression of (**A**) Caspase-9, (**B**) Caspase-3, (**C**) Bcl-2, and (**D**) AKT-1 genes in immunocompromised mice. Different lowercase letters indicate a significant difference between different groups (*p* < 0.05).

As shown in Figure 5, CTX stimulation significantly increased the expression levels of Caspase-9 and Caspase-3 genes in the thymus of mice (*p* < 0.05), while the expression of Bcl-2 showed a slight downward trend. FE intervention significantly decreased the expression levels of Caspase-9 and Bcl-2, even falling below the levels in the CON group, and had no significant effect on Caspase-3 expression (*p* ≥ 0.05). The intervention effects of DP and DPI groups on the expression levels of these three genes were significant, with levels being significantly reduced to the normal level of the CON group (*p* < 0.05). No significant differences were observed in the expression of AKT-1 gene in the thymus of each group of mice (*p* ≥ 0.05). These results suggested that DPI may mitigate the damage of CTX to thymic tissue by modulating the apoptotic pathway.

### 3.6. The Effect of DPI on Serum Cytokine Levels

**Figure 6:**
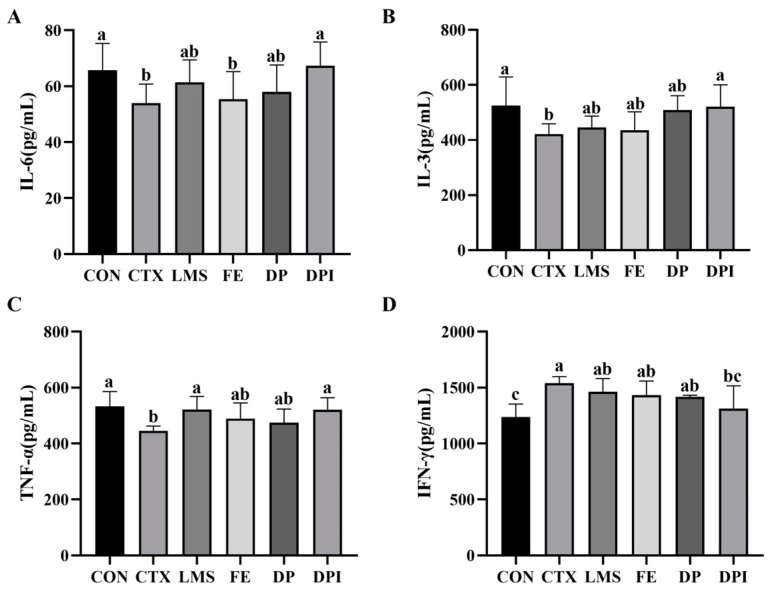
Cytokine levels in the serum of mice in each group. (**A**) IL-6, (**B**) IL-3, (**C**) TNF-α, and (**D**) IFN-γ. Different lowercase letters indicate a significant difference between different groups (*p* < 0.05).

The ELISA assay kit was used to measure and analyze the levels of relevant immune factors in the serum, as depicted in Figure 6. The CTX group showed significantly reduced levels of IL-6, IL-3, and TNF-α compared to the CON group (*p* < 0.05). The DPI group showed the most significant intervention effect (*p* < 0.05), with cytokine levels comparable to those in the CON group. In addition, the CTX group significantly increased IFN-γ levels compared to the CON group (*p* < 0.05), the intervention group showed a decline in IFN-γ, while only the DPI group showed a significantly reduced IFN-γ level (*p* < 0.05), although they had not yet returned to normal level. These results indicated that DPI can effectively restore the disrupted levels of immune cytokines disrupted by CTX, thereby enhancing immunity.

### 3.7. Metabolic Regulation of DPI on Immunocompromised Mice

#### 3.7.1. Multivariate Statistical Analysis

**Figure 7:**
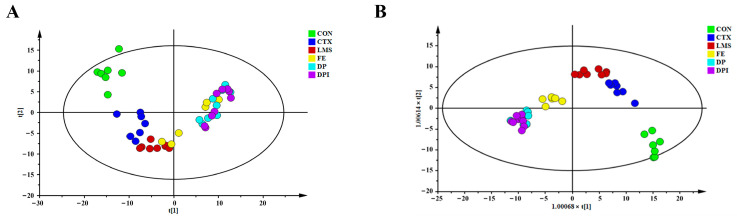
Pattern recognition analysis in positive ion modes. (**A**) PCA score plot; (**B**) OPLS-DA score plot.

In the positive ion mode, the PCA plot (Figure 7A) clearly delineated a grouping trend, with a distinct separation between the CON group and the CTX group, indicating the successful establishment of an immunosuppressive model. The distribution of the FE group was relatively scattered, suggesting great variability within the group. The distribution of the DP and DPI groups was relatively close, and distinct from the LMS and CTX groups. It was believed that the intervention of DP and DPI had an effect on improving immune suppression in CTX mice, and the mechanism of action was different from that of LMS. The OPLS-DA plot (Figure 7B) showed that each group was significantly separated with high-intra group correlation. The CON, LMS, FE, DP, DPI, and CTX groups were all distinctly separated, and there was a high degree of overlap between the DP and DPI groups. The DP and DPI groups demonstrated a better improvement effect on CTX, approaching the normal level of the CON group. The trends observed in PCA and OPLS-DA in the negative ion mode (Figure S1) were consistent with those in the positive ion mode, reflecting the metabolic intervention trend of DPI on immunocompromised mice.

**Figure 8:**
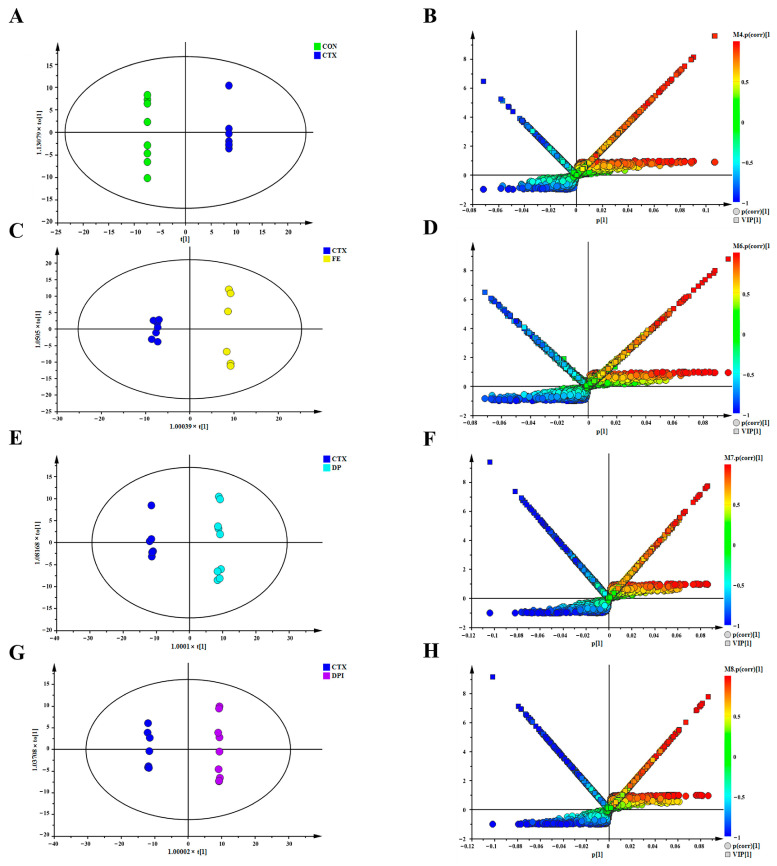
OPLS-DA diagram and (V + S) plot diagram of mouse plasma samples among the groups in positive ion mode. (**A**,**B**): OPLS-DA and (V + S) plots of CON and CTX groups; (**C**,**D**): OPLS-DA and (V + S) plots of CTX and FE groups; (**E**,**F**): OPLS-DA and (V + S) plots of CTX and DP groups; (**G**,**H**): OPLS-DA and (V + S) plots of CTX and DPI groups.

Pairwise comparisons were performed on plasma samples, supervised OPLS-DA modeling was performed, and potential differences in metabolites between the two groups were highlighted by combining VIP values and the S-plot. The OPLS-DA score and variable importance (V + S) plot plots for metabolic data between plasma sample groups under both positive and negative ion modes were obtained (Figure 8). In the positive ion mode, the CON group and CTX group were clearly separated in the OPLS-DA plot. Compared to the CON group, the CTX group showed significant differences in plasma characteristics, further confirming the successful induction of immune depression by CTX injection. The significant separation of the CTX group from the FE, DP, and DPI groups indicated their significant regulatory effects on plasma metabolism in CTX-induced immunosuppressive mice. In addition, OPLS-DA models were established for FE, DP, and DPI in pairs, but none of them passed the CV-ANOVA test (*p* ≥ 0.05), and complete separation could not be achieved among the three groups. It is believed that DP, FeSO_4_, and DPI may have overlapping regulatory effects in improving immune suppression, suggesting the possible existence of partially identical metabolic pathways. In the negative ion mode, the same plasma differential characteristics were observed in the OPLS-DA plot (Figure S2) between the groups, further demonstrating the significant metabolic regulatory effects of DP, DPI, and FeSO_4_ on immune suppression. The (V + S) plot in both positive and negative ion modes provided comprehensive information on endogenous differential metabolites between groups.

#### 3.7.2. Screening and Identification of Plasma Differential Metabolites

**Figure 9:**
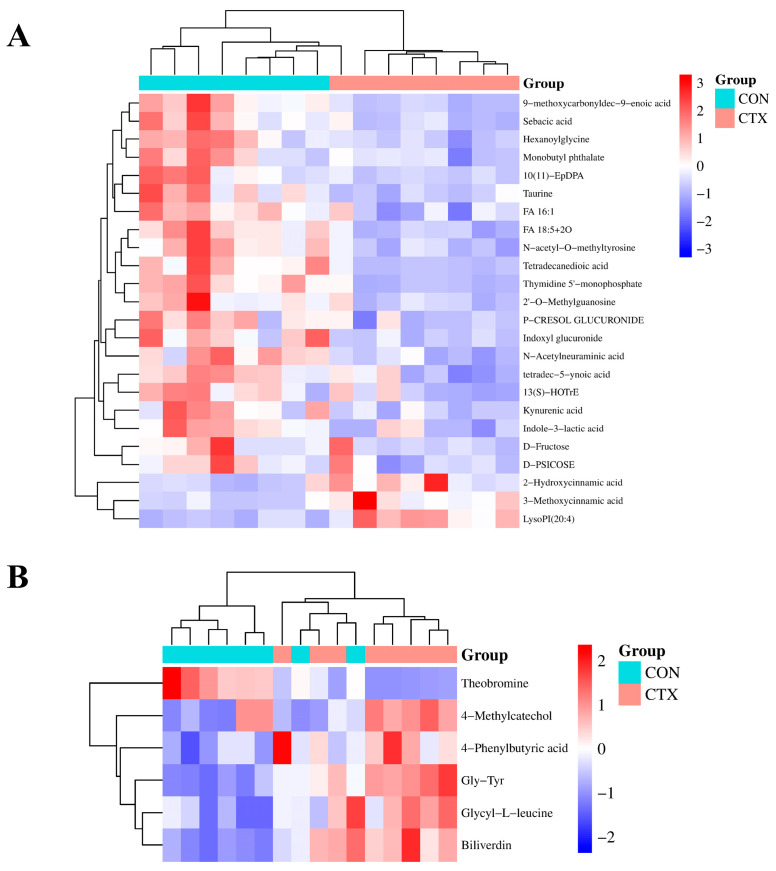
Hierarchical clustering heatmap of differential metabolites in positive ion (**A**) and negative ion (**B**) modes.

When VIP > 1.0, *p* (corr) > 0.6 (or < −0.6), and *p* < 0.05, it was considered that the screened metabolites were significantly endogenous differential metabolites between the two groups of samples [22]. After database matching, 30 differential metabolites were identified from the CON and CTX groups in both positive and negative ion modes (Figure 9). Compared to the CON group, after CTX induction, the levels of taurine, d-fructose, kynurenic acid, indole-3-lactic acid, indoxyl glucuronide, *N*-acetylneuraminic acid, thymidine 5′-monophosphate, and theobromine in mouse plasma significantly decreased (*p* < 0.05), while the levels of biliverdin, 4-methylcatechol, 3-methoxycinnamic acid, hexaacylglycine, and Gly-Tyr significantly increased (*p* < 0.05). The changing trends of these differential metabolites offered insights into the underlying mechanisms of CTX-induced immune depression.

Due to the similar metabolic profiles observed in pattern discriminant analysis, differential metabolites were identified between the FE, DP, DPI, and CTX groups to explore the common regulation of metabolite levels in the treatment groups when intervening in CTX-induced immunocompromised mice. In both positive and negative ion modes, the intervention in the FE, DP, and DPI groups regulated the levels of 64 differential metabolites (Tables S3 and S4). Among these, DP and DPI jointly regulated 20 types, FE and DPI jointly regulated 14 types, and the 3 group jointly regulated 10 types. The fluctuation trends in the levels of these differential metabolites were consistent, indicating a certain degree of commonality in metabolic regulation patterns. All three intervention groups significantly (*p* < 0.05) increased the levels of 4-hydroxycyclohexanecarboxylic acid, octanoic acid, inosine, hypoxanthine, adenine, caffeic acid, lysozyme phosphatidylinositol, and corticosterone. However, the levels of 3-phosphate glycerol reduced significantly (*p* < 0.05). The similarity in the regulatory trends of these differential metabolites suggested that the FE, DP, and DPI groups may intervene in the CTX group through partially identical metabolic or signaling pathways.

In addition, compared with the CTX group, FE alone regulated 15 differential metabolites, significantly reducing the content of pyruvate acid, galactaric acid, 3-indoxyl sulphate, γ-glutamylleucine, *p*-cresyl sulfate, and FA 13:3+1O (*p* < 0.05), while significantly increasing the levels of β-leucine, glycine, phenylacetylglycine, indoleacetaldehyde, and lysoPI (18:0/0:0) in plasma (*p* < 0.05). DP intervention in CTX alone regulated 14 differential metabolites, significantly reducing the content of 5,6-dihydrouracil, dUMP, cholic acid, eicosapentaenoic acid, and 15-oxoEDE (*p* < 0.05), while significantly increasing the content of pantothenic acid, 3-methylglutaconic acid, 4-hydroxy-6-methyl-2-pyrone, urocanic acid, and *N*-acetyl-L-carnosine (*p* < 0.05). DPI intervention in CTX separately regulated 14 differential metabolites, significantly reducing the content of citrulline, biotin, *N*-acetyl-L-carnosine, and phenylacetylglycine (*p* < 0.05), and significantly increasing the content of decanoic acid, mevalonic acid lactone, succinate semialdehyde, isobutyric acid, indoxyl glucuronide, mandelic acid, and salicylic acid (*p* < 0.05).

KEGG pathway enrichment analysis was conducted on the differential metabolites, and metabolic pathways with *p* < 0.05 and Impact > 0.01 were selected for comparative analysis (Table 3). For CTX-induced immunosuppressive mice, FE predominantly regulated amino acid metabolism and energy metabolism pathways, DP primarily regulated purine metabolism, pyrimidine metabolism, pantothenic acid, and coenzyme A biosynthesis, and DPI regulated purine metabolism pathways.

**Figure 10:**
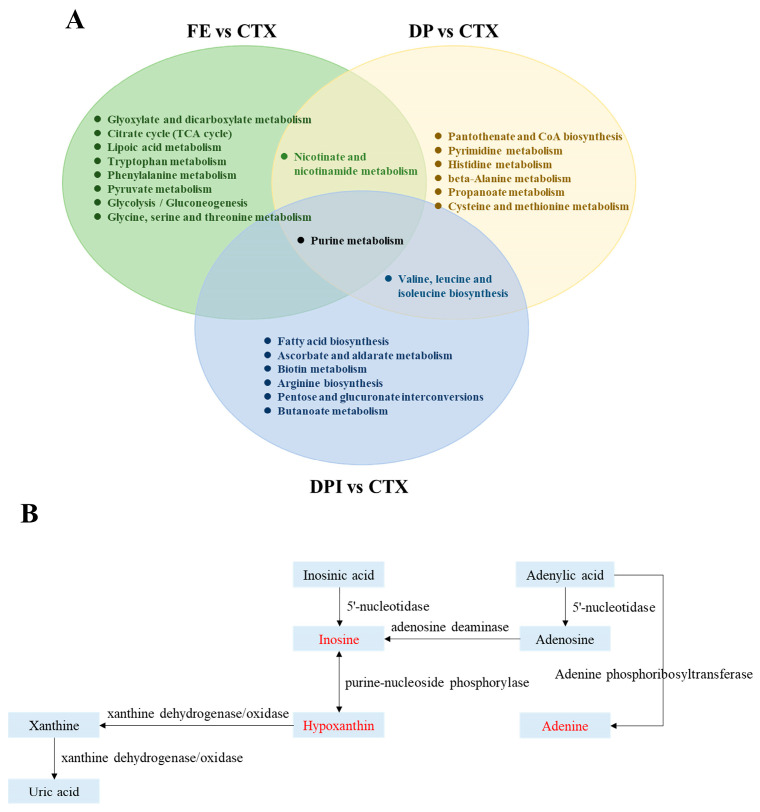
Metabolic pathway association analysis (**A**) and the potential metabolic pathway (**B**) of each intervention group.

Correlation analysis was conducted on the metabolic pathways regulated by FE, DP, and DPI in CTX mice to further explore the metabolic regulation patterns of these three groups in improving immunocompromised mice. As shown in Figure 10, the three groups jointly participated in the purine metabolism pathway by regulating the levels of inosine, adenine, and hypoxanthine, directly involving the degradation of adenine ribonucleotides and the pentose diphosphate pathway (nucleoside degradation). This suggested a potential pathway for DPI to alleviate immune suppression in the body.

#### 3.7.3. The Effect of DPI on Purine Metabolism Pathway in Immunocompromised Mice

**Figure 11:**
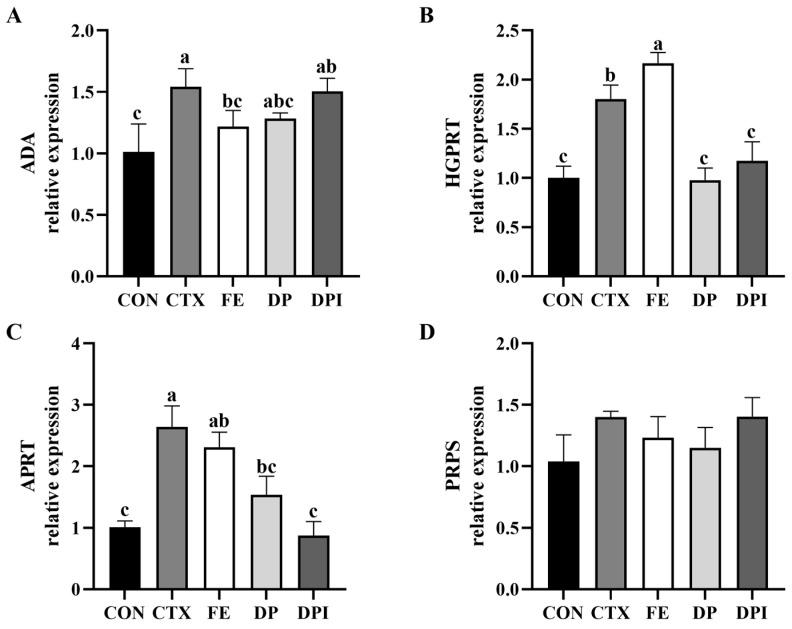
Effect of donkey bone collagen peptide iron chelate on purine metabolism related gene expression in immunocompromised mice. (**A**) ADA, (**B**) HGPRT, (**C**) APRT, and (**D**) PRPS. Different lowercase letters indicate a significant difference between different groups (*p* < 0.05).

The expression levels of purine metabolism-related genes in the thymus were measured, as shown in Figure 11. Compared with the CON group, the expression levels of ADA and APRT in the thymus of CTX-induced immunocompromised mice were significantly increased (*p* < 0.05), while the expression levels of HGPRT and PRPS were slightly increased but not significantly (*p* ≥ 0.05). Compared with the CTX group, FE significantly reduced the mRNA expression level of ADA (*p* < 0.05) and showed a downward trend in the expression of APRT and PRPS, but this trend was not significant. Additionally, the expression level of HGPRT in the FE group was also slightly upregulated (*p* ≥ 0.05). The DP group significantly reduced the expression levels of HGPRT and APRT (*p* < 0.05) and exhibited a certain regulatory ability for ADA and PRPS expression. DPI reduced the expression levels of HGPRT and APRT genes to normal levels, but it did not significantly regulate the expression of ADA and PRPS (*p* ≥ 0.05). These results indicated that DPI can regulate CTX-induced immune suppression by interfering with the purine metabolism pathway, with HGPRT and APRT as key targets in the regulatory pathway. By downregulating the expression of key genes, cytokine levels can be regulated, and immune cells can be protected.

#### 3.7.4. The Effect of DPI on the JAK/STAT Signaling Pathway in Immunocompromised Mice

**Figure 12:**
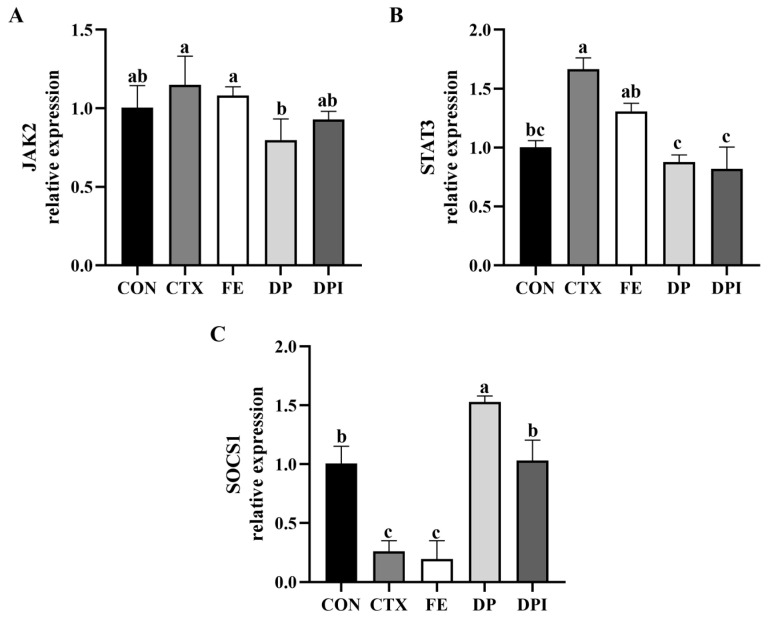
Effect of donkey bone collagen peptide iron chelate on the expression of JAK/STAT signaling pathway related genes in immunocompromised mice. (**A**) JAK2, (**B**) STAT3, and (**C**) SOCS1. Different lowercase letters indicate a significant difference between different groups (*p* < 0.05).

The expression levels of the JAK/STAT signaling pathway-related genes in the thymus were measured, as shown in Figure 12. Compared with the CON group, CTX significantly upregulated the STAT3 gene level in the thymus of mice (*p* < 0.05) and significantly downregulated the SOCS1 gene level (*p* < 0.05). The DP and DPI groups showed a significant intervention effect (*p* < 0.05). In addition, after CTX stimulation, there was no significant fluctuation in the JAK2 gene level (*p* ≥ 0.05), suggesting that this immunosuppressive process does not activate the STAT family through the JAK2 protein, and may involve other activation pathways.

## 4. Discussion

In recent years, more and more studies have been conducted on peptide metal chelates with immune functions. However, the immunomodulatory effects of these peptides and their metal chelating peptide immunomodulators are usually only verified from the perspective of immune organs, inflammatory responses, and intestinal mucosa, and few studies have examined their immunomodulatory mechanisms via the purine salvage pathway.

Additionally, research on donkey collagen peptide iron chelates has primarily focused on Ejiao, which has been shown to affect hematopoietic function and immune regulation. Donkey bone collagen, similar in amino acid composition and proportion to donkey skin, is abundant in type I collagen [23]. Its hydrolysates can cross the epithelium into the bloodstream as short peptides, demonstrating high bioavailability [24]. The presence of alkaline amino acids and hydrophobic residues, such as Arg, Pro, and Ala, contribute to its immune-stimulating properties [25]. Glu and Arg residues provide binding sites for Fe^2+^, aiding in the formation of peptide iron chelates that regulate hematopoietic and immune balance [11,26]. In this study, we validated the immunomodulatory effects of DP and DPI and analyzed the metabolic mechanism of DPI intervention in immune deficiency using a non-targeted metabolomics method based on UPLC–MS/MS, further validating the relevant metabolic and signaling pathways.

The immune system consists of central and peripheral immune organs, of which the central immune organs include the bone marrow and the thymus, while the peripheral immune organs include the lymph nodes and the spleen. The thymus and spleen are key organs of immune response, and their visceral indicators are often used as important indicators to evaluate immune homeostasis [27]. However, CTX causes significant damage to the immune system, particularly by impairing the hematopoietic environment of the bone marrow, leading to a short-term reduction in RBCs and anemia. In this case, the spleen would undergo compensatory extramedullary hematopoiesis, resulting in splenomegaly [28]. However, in this study, it was found that DPI could significantly reduce the spleen index (*p* < 0.05), indicating that DPI had a protective effect on spleen physiological function and the hematopoietic microenvironment. In addition, CTX can also cause hepatorenal toxicity, leading to oxidative damage of the liver and kidney [29]. Therefore, the regulatory effects of DP and DPI on organ indices reflected their potential in protecting immune and metabolic organs.

Bone marrow is distributed in different long bones, short bones, and flat bones, and is one of the largest organs in the human body [30]. Bone marrow is not only the main site of hematopoietic activity, but also plays a crucial role in storing and maintaining immune memory [31]. However, CTX can damage the bone marrow hematopoietic microenvironment, leading to bone marrow suppression and immune suppression. This disruption affects the normal production and function of hematopoietic progenitors, hematopoietic stem cells, and lymphocytes of different lineages [32]. H&E staining of femoral bone marrow tissue pathology images displayed that DPI, by restoring the level of megakaryocytes and Neu, improved the CTX-induced structural damage to bone marrow function.

The spleen is a typical peripheral immune organ, which is the main site of aggregation of mature T and B cells, and also the initiation site of immune response after encountering antigens. The proliferation of splenic lymphocytes is a key indicator of immune enhancement, reflecting the cellular immune ability of the body [33]. In immunosuppressive mice, spleen lymphocyte proliferation ability enhancement can activate the immune system, and then adjust the immune function [34]. In this study, the results of spleen lymphocyte proliferation assay from each intervention group showed that DPI could enhance spleen lymphocyte proliferation and improve cellular immune function, thereby reducing immune organ damage.

Studies have shown that CTX significantly affected the formation of antibodies, WBCs, and Lym, thereby inducing immunosuppression [35]. In addition, CTX can also damage bone marrow cells, leading to impaired hematopoietic function, reduced red blood cell levels, and even anemia [36]. Due to the short half-lives of WBCs, they are key cells sensitive to immune system stimulation, with levels that fluctuate significantly. Therefore, we evaluated the severity of immunosuppression caused by CTX based on WBC level. The study found that DPI significantly reduced the levels of WBCs and Bas (*p* < 0.05), alleviating the imbalance of immune cells caused by CTX. Bas are the main effector cells participating in allergic reactions; in the immune response, they release histamine, which can activate helper T cells to suppress cytotoxic T cells. Fe^2+^ and DPI significantly decreased the level of these cells (*p* < 0.05), which may be related to iron or peptide iron chelate intervention in helper T cell activation, thereby restoring cytotoxicity [37]. For other immune cells, the regulatory effects of each intervention group were not significant, which may be due to the direct killing of the lymphocyte population by CTX by inhibiting lymphocyte proliferation and secretion [38], leading to the disturbance of immune cell proportion and immunosuppression. Due to the mild nature of immune modulators, they are unable to completely reverse immunosuppression for other immune cells.

In the context of immune system homeostasis, the viability of thymic cells serves as a critical indicator reflecting their equilibrium state. Disruption of this balance may lead to profound dysregulation of the body’s defense mechanisms [39]. The Caspase family of cell apoptosis proteases plays a pivotal role in finely regulating multiple stages of the cell apoptosis process through cascade reactions and specific substrate cleavage, exerting precise control over this pathway [40]. Specifically, Caspase-3 acts as the ultimate executor in the cascade reaction of cell apoptosis, playing a decisive role in its terminal stage [41]. The activation of Caspase-9 involves both exogenous and endogenous pathways, with the former primarily mediated by death ligands and the latter closely associated with mitochondrial pathways [42]. The Bcl-2 protein, functioning as a key anti-apoptotic molecule, protects cells from apoptosis by inhibiting mitochondrial release of cytochrome C and activation of caspase enzymes. In addition to these functions, it prevents ONOO- formation [43]. Within the complex process of cellular apoptosis, acrolein—a metabolic product derived from CTX—promotes intracellular ROS and NO generation. This subsequently increases the ONOO- concentration, which activates apoptotic enzyme systems, leading to lipid peroxidation, protein oxidation, DNA strand breaks, disruption in mitochondrial function. This ultimately results in the loss of mitochondrial membrane potential and the release of cytochrome C, activating Caspase family proteins and triggering cellular damage and apoptosis via an endogenous pathway [44,45,46]. DPI is considered a potential protective agent that may mitigate intracellular oxidative stress, thereby safeguarding mitochondrial function. Consequently, DPI regulates expression levels related to genes involved in apoptosis, ultimately providing protection for cells.

Cytokines play a crucial role in regulating host immune responses to infection, inflammation, immunity, and cancer [47]. Firstly, IL-6 is mainly involved in humoral immunity and is associated with organ damage and bone marrow suppression caused by CTX [48]. Secondly, IL-3 is associated with megakaryocyte generation, reflecting the homeostasis of the hematopoietic microenvironment [49]. In addition, TNF-α can initiate innate immune responses, enhance the activity of T and B lymphocytes, and interact with other inflammatory cytokines [50]. Finally, IFN-γ is mainly involved in the cellular immune process by activating macrophages and enhancing antigen presentation [50]. In this study, CTX significantly affected the level of cytokines (*p* < 0.05), while DPI showed a significant intervention effect, indicating that it was involved in various immune response pathways, promoting the function of multiple immune cells.

Metabolomics technology provides a visual analysis tool for exploring the metabolic characteristics of immunosuppressed mice after DPI intervention. By exploring potential differential metabolites and metabolic pathways, we further demonstrated the metabolic modulatory effects of DPI on CTX-induced immunosuppression in mice [51]. First of all, the FE group adjusted the energy metabolism of immunosuppressive mice induced by CTX. The disorder of TCA metabolism caused by immune stimulation inhibited isocitrate dehydrogenase and succinate dehydrogenase (SDH), weakened TCA metabolism, and, thus, limited the immune function of macrophages [52]. However, the introduction of iron improved the steady state of Fe-S clusters and promoted the generation of SDH [53]. In addition, DPI also adjusted arginine biosynthesis. Arginine participates in the production of a variety of amino acids in the body and regulates the levels of inflammatory factors by affecting the urea cycle, proline metabolism, and glutamate transamination, while protecting immune organs and promoting the proliferation and differentiation of T lymphocytes [54]. Peptide intervention of DPI increased intake of proline, while promoting iron intake by the TCA cycle was conducive to regulating immune cytokine release in the body.

The co-regulation of purine metabolism by the FE, DP, and DPI groups through the same locus revealed the potential of DPI to alleviate immunosuppression. Purine metabolism refers to the synthesis and decomposition of purine derivatives, such as adenine and guanine, in the body. As a key enzyme in purine metabolism, adenosine deaminase (ADA) is mainly involved in the degradation of adenine ribonucleotide, which is also an important enzyme in purine rescue pathway [55]. ADA can affect the proliferation of T lymphocytes and the production of cytokines, thereby participating in the maintenance of cellular immunity [56]. Additionally, HGPRT is also involved in the purine salvage pathway, and its expression is negatively correlated with the expression of genes related to immune function [57]. When CTX caused the direct killing of cells in immune organs, the proliferation of lymphocytes led to an increased energy demand and, thus, an increased demand for adenosine nucleotides, resulting in the upregulation of APRT expression [58]. DPI’s regulation of these three enzymes indicated that DPI primarily interfered with the purine salvage pathway to modulate CTX-induced immunosuppression, with HGPRT and APRT being key targets in this regulatory pathway. By downregulating the expression of these key genes, DPI can regulate cytokine levels, thereby achieving the protection of immune cells.

In addition, the JAK/STAT pathway is an important cascade involved in multiple growth factors and cytokine signaling, which regulates gene expression, cell activation, proliferation, and differentiation [59]. Specifically, the negative regulatory signaling cascade of SOCS1 is closely related to the STAT3 pathway [60]. When CTX stimulation occurred, it decreased SOCS1 expression and activated the STAT3 pathway, thereby participating in the immunosuppressive process. This may be related to the need for tissue repair following CTX-induced cell death. The expression of the STAT3 gene correlated with the release of the IL-6 signaling factor and, to some extent, reflected the differentiation ability of CD4 T cells [61]. Consequently, after CTX stimulation, the expression of STAT3 was significantly increased (*p* < 0.05). However, DPI reversed the expression levels of these two genes, adjusted the activation of the JAK/STAT signaling pathway, and thereby demonstrated the potential to protect cells and promote T lymphocyte proliferation and differentiation.

## 5. Conclusions

DP and DPI from immune organs, immune cells, and immune factors reduce the immune deficiency induced by CTX. DPI significantly enhanced the physiological status of CTX-induced immunocompromised mice by adjusting blood count parameters, primarily WBCs, and promoting splenic lymphocyte proliferation similarly to DP. Additionally, DPI mitigated CTX-induced cell apoptosis by regulating Caspase-9, Caspase-3, and Bcl-2 genes, effectively mitigating the killing effect of CTX on lymphocytes in immune organs. DPI also significantly reduced organ index fluctuations caused by CTX, demonstrating a protective effect on bone marrow tissue structure. Furthermore, DPI showed a significant intervention effect on CTX-induced abnormal immune factor levels. Fe^2+^, DP, and DPI all significantly regulated inosine, hypoxanthine, and adenine levels, indicating involvement in purine metabolic pathways. FE and DP separately regulate energy metabolism and pyrimidine metabolism pathway. DPI primarily regulated the purine salvage pathway through key targets HGPRT and APRT, and also modulated the JAK/STAT signaling pathway via STAT3 and SOCS1 genes, demonstrating a protective effect on immune cells and a restorative effect on immune factor levels. These findings provide a theoretical basis for the further development of functional products derived from donkey collagen peptides.

## Figures and Tables

**Table 1:** Gene primer sequence for RT-qPCR.

Gene	Forward Primer (5′-3′)	Reverse Primer (5′-3′)
β-actin	GGCTGTATTCCCCTCCATCG	CCAGTTGGTAACAATGCCAT
ADA	CCCAGACACCCGCATTCAAC	CGATGCCTCTCTTCTTGCCAAA
HGPRT	TCAGTCAACGGGGGACATAAA	GGGGCTGTACTGCTTAACCAG
APRT	CCCTCTTGAAAGACCCGGAC	TCCAGAGAATAGGAGGCTGAC
PRPS	ATGCCTAACATCGTGCTCTTC	GATCTCGACACTGGTCTCCTG
Caspase-9	TCCTGGTACATCGAGACCTTG	AAGTCCCTTTCGCAGAAACAG
Caspase-3	ATGGAGAACAACAAAACCTCAGT	TTGCTCCCATGTATGGTCTTTAC
Bcl-2	ATGCCTTTGTGGAACTATATGGC	GGTATGCACCCAGAGTGATGC
AKT-1	ATGAACGACGTAGCCATTGTG	TTGTAGCCAATAAAGGTGCCAT
JAK2	CTTGTGGTATTACGCCTGTGT	TGCCTGGTTGACTCGTCTATG
STAT3	CAATACCATTGACCTGCCGAT	GAGCGACTCAAACTGCCCT
SOCS1	CTGCGGCTTCTATTGGGGAC	AAAAGGCAGTCGAAGGTCTCG

**Table 2:** Basic hemogram indexes of mice in each group on the third day of inducing (*n* = 6, mean ± SD).

Group	WBCs (10^9^/L)	RBCs (10^9^/L)	PLT (10^9^/L)
CON	8.4075 ± 1.93 ^a^	11.4060 ± 0.24 ^a^	754.6000 ± 39.50
CTX	3.3240 ± 0.80 ^b^	10.4440 ± 0.51 ^b^	703.8000 ± 127.95
LMS	3.2180 ± 0.28 ^b^	10.1200 ± 0.60 ^b^	682.2000 ± 31.01
FE	4.1350 ± 0.74 ^b^	10.3800 ± 0.84 ^b^	711.0000 ± 73.62
DP	3.4625 ± 0.26 ^b^	10.3560 ± 0.20 ^b^	764.8000 ± 62.19
DPI	3.7440 ± 0.50 ^b^	9.9300 ± 0.69 ^b^	702.2000 ± 60.52

Different lowercase letters indicate a significant difference between different groups (*p* < 0.05).

**Table 3:** Differential metabolic regulation pathways of mice in each group.

Group	Pathway	*p* Value	Impact
FE vs. CTX	Glycine, serine, and threonine metabolism	0.0194	0.28464
	Tricarboxylic acid (TCA) cycle	0.0069	0.09637
	Dicarboxylic acid metabolism	0.0010	0.12963
DP vs. CTX	Purine metabolism	0.0242	0.0416
	Pantothenic acid and coenzyme A biosynthesis	0.0135	0.013501
	Pyrimidine metabolism	0.0477	0.13221
DPI vs. CTX	Purine metabolism	0.0197	0.0416

## Data Availability

The original contributions presented in the study are included in the article/Supplementary Materials; further inquiries can be directed to the corresponding author.

## Supplementary Materials

The following supporting information can be downloaded at: https://www.mdpi.com/article/10.3390/nu16152413/s1, Figure S1: Pattern recognition analysis in negative ion modes. (A) PCA score plot; (B) OPLS-DA score plot. Figure S2: OPLS-DA diagram and (V + S) plot diagram of mouse plasma samples among groups in negative ion mode. Table S1: Chromatographic mobile phase. Table S2: UPLC–MS/MS mobile phase elution procedure. Table S3: Significant metabolic markers of mice plasma samples in the negative ion mode. Table S4: Significant metabolic markers of mice plasma samples in the positive ion mode.
