# Supplementary material for: Interventional Effect of Donkey Bone Collagen Peptide Iron Chelate on Cyclophosphamide Induced Immunosuppressive Mice
Source: Nutrients. 2024 Jul 25;16(15):2413. doi: 10.3390/nu16152413 (PMC11314553; doi:10.3390/nu16152413)
Supplement: Supplementary file 1 [file nutrients-16-02413-s001.zip › Supplementary Material.pdf]

Supplementary Material

Table S1. Chromatographic mobile phase

Mobile phase	Positive ion mode	Negative ion mode
Mobile phase A	Methanol solution containing 0.1% formic acid	Methanol solution containing 0.05% acetic acid
Mobile phase B	An aqueous solution containing 0.1% formic acid and 5% methanol	An aqueous solution containing 0.05% acetic acid and 5% methanol

Table S2. UPLC-MS/MS mobile phase elution procedure

Time（min）	Mobile phase A（%）	Mobile phase B（%）	Velocity of flow（mL/min）
0	0	100	0.35
1.0	0	100	0.35
5.5	100	0	0.35
8.0	100	0	0.35
8.1	0	100	0.35
12.0	0	100	0.35

Table S3. Significant metabolic markers of mice plasma samples in the negative ion mode

No	Metabolites	RT/min	m/z	FE vs CTX		DP vs CTX		DPI vs CTX	
				FC	Change	FC	Change	FC	Change
1	4-Hydroxycyclohexanecarboxylic acid	0.535	143.070	1.130	↑**	1.204	↑**	1.208	↑**
2	Caprylic acid	0.546	143.106	1.253	↑*	1.392	↑**	1.434	↑**
3	Decanoic acid	0.571	171.138					1.201	↑**
4	Mevalonic acid lactone	0.594	129.054					1.236	↑**
5	5,6-Dihydrouracil	0.821	113.034			0.896	↓*		
6	Citrulline	0.856	174.087					0.729	↓**
7	6-Deoxy-L-galactose	0.895	163.060			0.877	↓*	0.877	↓*
8	Ascorbic acid	0.91	175.024			0.507	↓*	0.497	↓**
9	Pyruvic acid	0.923	87.007	0.145	↓*				
10	trans-Aconitic acid	0.925	173.008			0.674	↓**	0.652	↓**
11	Inosine	0.938	267.073	2.463	↑*	2.845	↑**	2.713	↑**
12	Hypoxanthine	0.972	135.030			1.435	↑*	1.541	↑**
13	Glycerol 3-phosphate	0.976	171.005	0.547	↓*	0.457	↓*	0.538	↓**
14	Galactaric acid	0.996	209.029	0.920	↓*				
15	2-Oxobutyric acid	1.018	101.023			0.056	↓**	0.045	↓**
16	cis-Aconitic acid	1.064	173.008	0.805	↓*				
17	3-Hydroxy-3-methylglutaric acid	1.104	161.044	1.297	↑**	1.527	↑*	1.479	↑*
18	dUMP	1.125	307.034			0.804	↓**		
19	Succinate semialdehyde	1.238	101.023					2.705	↑**
20	β-Leucine	1.27	130.086	1.265	↑**				

21	Isobutyric acid	1.788	87.044					1.380	↑**
22	2',3'-Dideoxyinosine	1.873	235.082					1.133	↑*
23	Pantothenic acid	2.579	218.103			1.476	↑**		
24	3-Methylglutaconic acid	2.694	143.034			1.387	↑*		
25	Indoxyl glucuronide	2.895	308.077					1.120	↑**
26	3-Indoxyl sulphate	2.941	212.001	0.400	↓*				
27	Valeric acid	2.948	101.059			1.734	↑**	1.647	↑**
28	4-Hydroxy-6-methyl-2-pyrone	3.000	125.023			1.234	↑**		
29	γ-Glutamylleucine	3.157	259.130	0.723	↓*				
30	Mandelic acid	3.244	151.039					1.328	↑*
31	Salicylic acid	3.247	137.023					1.219	↑**
32	2,3-Dihydroxybenzoic acid	3.426	153.018			1.135	↑*	1.141	↑**
33	Biotin	3.427	243.080					0.902	↓**
34	Glycine	3.431	74.023	1.079	↑*				
35	Phenylacetylglycine	3.432	192.066	1.552	↑*				
36	Benzoic acid	3.497	121.028					1.737	↑**
37	p-Cresol glucuronide	3.643	283.082	2.228	↑**			1.564	↑*
38	FA 9:2+1O	3.648	169.086					1.354	↑*
39	4-Methylphenol	3.657	107.049	1.295	↑**				
40	p-Cresol sulfate	3.659	187.006	0.352	↓*				
41	Caffeic acid	3.826	179.034	1.142	↑**	1.194	↑**	1.168	↑**
42	Indoleacetaldehyde	3.829	158.060	1.075	↑*				
43	N-acetyl-O-methyltyrosine	4.026	236.092	0.897	↓*	0.872	↓*	0.854	↓**
44	FA 9:1+1O	4.223	171.102	1.231	↑*			1.176	↑**
45	N-Acetyl-L-carnosine	5.460	267.114			1.142	↑*		
46	Cholic acid	5.734	407.280			0.757	↓*		
47	FA 13:3+1O	5.806	223.133	0.885	↓*				
48	LysoPI(18:0/0:0)	5.900	599.319			0.673	↓*	0.650	↓**
49	Eicosapentaenoic acid	6.014	301.217			0.909	↓*		
50	15-OxoEDE	6.072	321.243			0.857	↓**		
51	FA 19:1	6.565	295.264	0.842	↓*				
52	Urocanic acid	7.003	137.034			1.253	↑**		
53	LysoPI(20:4)	8.926	619.289	1.579	↑**	1.668	↑**	1.638	↑**
54	LysoPI(16:0/0:0)	9.047	571.289			0.868	↓**	0.847	↓**

FC: Fold change, representing the multiple changes in differential metabolite levels between the intervention group and the CTX group; ↑: The intervention group upregulated differential metabolite levels compared to the CTX group; ↓: The intervention group downregulated differential metabolite levels compared to the CTX group; *: Significant changes (P<0.05); * *: The changes were extremely significant (P<0.01). Same below.

Table S4. Significant metabolic markers of mice plasma samples in the positive ion mode

No	Metabolites	RT/min	m/z	FE vs CTX		DP vs CTX		DPI vs CTX	
				FC	Change	FC	Change	FC	Change

1	Acetyl-L-carnitine	1.078	204.1225					0.861	↓**
2	Acetoacetate	1.119	140.9956					0.847	↓**
3	Inosine	1.444	291.0687			1.914	↑*		
4	Hypoxanthine	1.454	137.0453			2.642	↑*		
5	3-Hydroxyanthranilic acid	1.481	154.0494	154.049	↑**			1.355	↑**
6	Nudifloramide	1.665	153.0654	1.225	↑*	1.192	↑**	1.205	↑**
7	Adenine	1.833	136.0614	1.608	↑*	1.409	↑**	1.441	↑*
8	Corticosterone	5.084	347.2202	347.220	↑**	1.685	↑**	1.672	↑**
9	N-Acetylglutamine	5.440	189.0855			1.107	↑**		
10	Glycyl-L-leucine	6.191	189.1267	0.904	↓*				
11	alpha-Humulene	6.639	205.1942	1.190	↑*				
12	4-Phenylbutyric acid	7.010	165.0905			0.833	↓*		

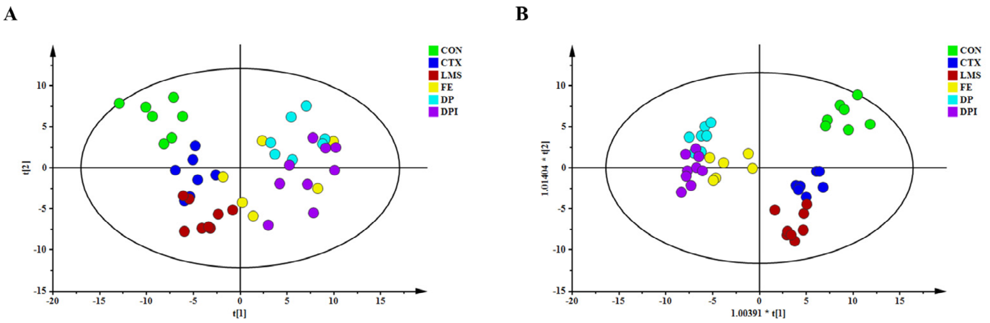

Figure S1. Pattern recognition analysis in negative ion modes. (A) PCA score plot, (B) OPLS-DA score plot.

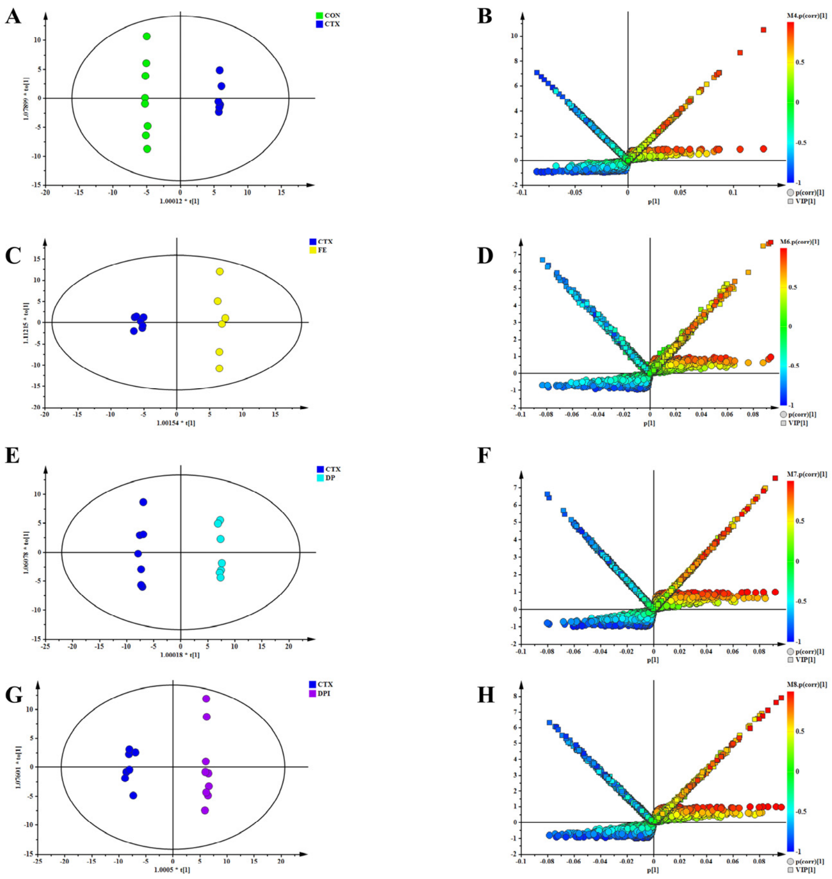

Figure S2. OPLS-DA diagram and (V+S) -plot diagram of mouse plasma samples among groups in negative ion mode. (A) and (B): OPLS-DA and (V+S) - plot plots of CON and CTX groups; (C) and (D): OPLS-DA and (V+S) - plot plots of CTX and FE groups; (E) and (F): OPLS-DA and (V+S) - plot plots of CTX and DP groups; (G) and (H): OPLS-DA and (V+S) - plot plots of CTX and DPI groups.
